# The Interaction between Factor H and Von Willebrand Factor

**DOI:** 10.1371/journal.pone.0073715

**Published:** 2013-08-26

**Authors:** Shuju Feng, Xiaowen Liang, Miguel A. Cruz, Hangoc Vu, Zhou Zhou, Naresh Pemmaraju, Jing-Fei Dong, Michael H. Kroll, Vahid Afshar-Kharghan

**Affiliations:** 1 Division of Internal Medicine, Benign Hematology, University of Texas, M. D. Anderson Cancer Center, Houston, Texas, United States of America; 2 Institute of Biosciences and Technology, Texas A&M University Health Science Center, Houston, Texas, United States of America; 3 Cardiovascular Research Section, Baylor College of Medicine, Houston, Texas, United States of America; 4 Puget Sound Blood Center, Seattle, Washington, United States of America; Institut National de la Santé et de la Recherche Médicale, France

## Abstract

Complement factor H (fH) is a plasma protein that regulates activation of the alternative pathway, and mutations in fH are associated with a rare form of thrombotic microangiopathy (TMA), known as atypical hemolytic uremic syndrome (aHUS). A more common TMA is thrombotic thrombocytopenic purpura, which is caused by the lack of normal ADAMTS-13-mediated cleavage of von Willebrand factor (VWF). We investigated whether fH interacts with VWF and affects cleavage of VWF. We found that factor H binds to VWF in plasma, to plasma-purified VWF, and to recombinant A1 and A2 domains of VWF as detected by co-immunoprecipitation (co-IP) and surface plasmon resonance assays. Factor H enhanced ADAMTS-13-mediated cleavage of recombinant VWF-A2 as determined by quantifying the cleavage products using Western-blotting, enhanced cleavage of a commercially available fragment of VWF-A2 (FRETS-VWF73) as determined by fluorometric assay, and enhanced cleavage of ultralarge (UL) VWF under flow conditions as determined by cleavage of VWF-platelet strings attached to histamine stimulated endothelial cells. Using recombinant full-length and truncated fH molecules, we found that the presence of the C-terminal half of fH molecule is important for binding to VWF-A2 and for enhancing cleavage of the A2 domain by ADAMTS-13. We conclude that factor H binds to VWF and may modulate cleavage of VWF by ADAMTS-13.

## Introduction

Factor H (fH) is a plasma protein that negatively regulates the alternative complement pathway in both fluid phase and on cell surfaces. It has a molecular mass of 150kD and circulates in plasma at a concentration of about 500 µg/ml (3 µM). Factor H prevents propagation of complement activation by promoting cleavage of C3b by plasma factor I (cofactor activity). Factor H is composed of 20 homologous structural domains, known as short consensus repeats (SCR). Despite the structural similarities between different SCRs, there are functional distinctions between different regions of fH. The N-terminal SCRs 1-4 are necessary for cofactor activity [1,2], whereas C-terminal SCRs 18-20 are responsible for binding of fH to the cell surface and regulating complement activity on the cell surface [3,4].

Mutations in the factor H gene are associated with a rare familial form of thrombotic microangiopathy known as atypical hemolytic uremic syndrome (aHUS) [5–10]. Most of these mutations are clustered in the C-terminal SCRs 18-20 of fH and result in synthesis of abnormal fH [5,9,10]. The mechanism linking abnormal function of fH to thrombotic microangiopathy in aHUS is not clear. Many of the mutant fH molecules are unable to bind to cell surfaces and control complement activation, resulting in complement-induced endothelial injury and platelet activation [11,12]. Interestingly, in animal studies, a total lack of fH was not associated with thrombotic microangiopathy [13], while expression of a truncated fH lacking the 5 C-terminal SCRs (SCRs 16-20) generated a phenotype very similar to that of aHUS [14]. In these transgenic mice, truncated fH maintained a near normal C3 concentration in plasma compared to a severely reduced C3 concentration in fH deficient mice.

Clinical manifestations of aHUS are similar to another thrombotic microangiopathy known as thrombotic thrombocytopenic purpura (TTP) which is caused by a decrease in the function of VWF-cleaving protease ADAMTS-13 (*A* Disintegrin And Metalloproteinase with a ThromboSpondin type 1 motif). Although aHUS is principally a kidney disorder and TTP is a more systemic disorder, often there is not a clear distinction between TTP and aHUS, especially in younger patients with a relapsing course. In typical HUS caused by Shiga toxin-producing bacteria the activity of ADAMTS-13 is within normal range, but there are few reports showing low activities of ADAMTS-13 in congenital relapsing HUS [15,16]. This observation raises the possibility of an etiologic link between aHUS and TTP resulting in clinical overlap between these two thrombotic microangiopathies. We hypothesized that fH’s role in the cleavage of VWF might connect etiologies of TTP and aHUS. We studied the physical interaction between fH and VWF and mapped the binding site of VWF on fH and, vice versa. Next we studied the effect of fH on ADAMTS-13-mediated cleavage of VWF and determined the region in fH involved in this process.

## Experimental Procedures

### Reagents

The ADAMTS-13 activity assay kit (Gen-Probe), plasma purified human factor H and factor I (Complement Technology Inc.), human factor H cDNA in pCMV6-XL4 vector (OriGene Technologies Inc.), plasma purified human VWF (Calbiochem), human VWF cDNA in pcDNA 3.1 vector, human ADAMTS-13 cDNA in pSectag vector (Invitrogen) [17,18], goat anti-human factor H and C3 (Complement Technology Inc.), monoclonal anti-human fH (clone 90X, Quidel), rabbit anti-human VWF (Dako), rabbit anti-GST (Invitrogen), goat anti-ADAMTS-13 (Novus Biologicals) antibodies, control normal goat and rabbit IgG (Santa Cruz Biotechnology), secondary antibodies including anti-goat (Bethyl) and anti-rabbit (Amersham Biosciences), and horseradish peroxidase-conjugated antibodies were purchased from the indicated sources.

### Washed platelets

Whole blood was collected into acid-citrate dextrose (ACD) anticoagulant (85 mM sodium citrate, 111 mM glucose, and 71 mM citric acid) at a 9: 1 vol/vol ratio, and centrifuged at 150 × g for 15 min (room temperature) to obtain platelet-rich plasma (PRP). To isolate platelets, PRP was centrifuged at 900 × g for 10 min, platelet pellets were washed, and resuspended in Tyrode buffer (138 mM sodium chloride, 5.5 mM glucose, 12 mM sodium bicarbonate, 2.9 mM potassium chloride, and 0.36 mM dibasic sodium phosphate, pH 7.4).

### Generating recombinant proteins

Full-length wild type human fH (F4 with SCRs 1-20, amino acids M^1^-R^1231^) and truncated fH (F1 with SCRs 1-5, amino acids M^1^-K^323^; F2 with SCRs 1-10, amino acids M^1^-E^625^; and F3 with SCRs 1-15, amino acids M^1^-G^928^) were generated by polymerase chain reaction (PCR) with 5′ primer: GGACTTTCCAAAATGTCGT, and 3′ primers (F1: ATCCTCGAGCTTTCAAGGTACATCT, F2: TTGCTCGAGCCTCTTTACATATTGG, F3: ACACTCGAGCGCCTTCACACTGAGG, and F4: ATCCTCGAGCTCTTTTTGCACAAGT), using human fH cDNA as a template. After confirming their sequence, the amplified fragments were digested with NotI (5′) and XhoI (3′) and ligated to pSecTag2B His tag vector (Invitrogen). The plasmids containing full-length or truncated factor H were transfected into mammalian HEK 293 cells using micelle carriers (Lipofectamine^TM^ 2000, Invitrogen). Stably transfected HEK 293 cells were maintained in Dulbecco’s Modified Eagle media containing 10% fetal bovine serum and the selection agent Hygromycin B (500µg/ml, Invitrogen). ADAMTS-13 cDNA was cloned into pSecTag2B His-tag vector (Invitrogen) at the HindIII and XbaI sites, and transfected into HEK 293 cells (Invitrogen) with Lipofectamine 2000 (Invitrogen). The recombinant proteins secreted into the serum-free medium were purified on a Ni-NTA column (Qiagen) and quantified using a BCA protein assay kit (Pierce).

The DNA sequence encoding the VWF A2 domain (amino acids P^1480^-G^1672^) was PCR amplified from the VWF cDNA using 5′ primer: ACTGGATCCCCGGGGCTCTTGGGG, and 3′ primer: CAGGAATTCTCCGGAGCAGCACCT, and ligated into pGEX-2T GST vector (GE Healthcare) or pSecTag2B His-tag vector. GST-A2 recombinant protein was expressed in *E. coli* and purified by chromatography on glutathione agarose beads (Invitrogen). His-tagged A2 was expressed in HEK 293 cells and purified by chromatography using Ni-NTA. Plasmids encoding His-tagged VWF-A1 domain (amino acids Q^1238^-D^1471^), VWF-A2 (amino acids G^1481^-R^1668^), and VWF-A3 domain (amino acids S^1671^-G^1874^) [19–21] were transfected to *E. coli*. The recombinant VWF-A1, VWF-A2, and VWF-A3 were purified by chromatography on Ni-NTA. A DNA fragment encoding the B and C domains of human VWF was amplified from VWF cDNA, subcloned into the pcDNA3.1/myc-His vector (Invitrogen), and transfected into HEK 239 cells. Myc-tagged VWF BC domains (VWF-BC) were purified using monoclonal Myc antibody (Invitrogen).

### Immunoprecipitation and Western blotting

Immunoprecipitations were performed by incubating plasma-purified factor H (30 and 60 nM) with plasma-purified VWF (20 nM) in the presence of rabbit anti-VWF antibody (10 µl) or control rabbit IgG. Sepharose-conjugated Protein A/G (20 µl) was added to each sample, and incubated overnight at 4^o^C. To study the association between fH and VWF in serum, 250 µl of normal serum was incubated with sepharose beads-conjugated protein A/G (protein A/G beads) for 2 hr at room temperature and centrifuged at 10,000 × *g*. The serum supernatant was incubated with either 10 µg anti-VWF antibody or control rabbit IgG, and protein A/G beads for another 2 hours. Sepharose-bead-captured proteins were washed with ice-cold PBS buffer containing 0.01% Triton-100 (PBS-T), separated by SDS-polyacrylamide gel electrophoresis (PAGE) under reducing conditions, transferred to immobilon-P membranes (Millipore), and detected by a goat anti-Factor H antibody (1:20,000, overnight at 4^o^C) and a peroxidase-conjugated rabbit anti-goat antibodies (1:40,000). Immunoprecipitations were also carried out by incubating plasma-purified factor H (30 nM) with recombinant VWF-A1 (100 nM), VWF-A2 (100 nM, purified from transfected *E. coli*), VWF-A2 (100 nM, purified from transfected HEK 293 cells), VWF-A3 (100 nM), or VWF-BC domains. In another group of experiments, recombinant GST-VWF-A2 (100 nM) or VWF-A1 (100 nM) was incubated with recombinant full-length or truncated fH (F4, F3, F2, or F1,30 nM each). After adding glutathione-conjugated agarose beads (20 µl) (for samples with GST-VWF-A2) or sepharose beads coated with polyclonal anti-VWF antibody (for samples with VWF-A1), each sample was incubated overnight at 4^o^C, and washed for three times. The precipitated proteins were separated by SDS-PAGE under reducing conditions. Proteins were transferred to membranes, immunoblotted with polyclonal or monoclonal anti-fH antibody, and visualized by chemiluminescence. The monoclonal anti-fH antibody (Clone 90X, Quidel Inc) recognizes an epitope in the SCR1 of fH.

To study C3b binding, recombinant fH fragments (F4, F3, F2, or F1,30 nM each) were incubated with C3b (70 nM). C3b bound proteins were immunoprecipitated using anti-C3 antibody (10 µg) and protein A/G beads (20 µl) and immunoblotted using an anti-fH antibody.

### Cofactor activity

The effect of recombinant fH fragments on factor I-mediated cleavage of C3b was studied by incubating 0.8 µM of plasma-purified fH, recombinant F1, F2, F3, or F4 with 0.7 µM of C3b and 0.5 µM of plasma-purified factor I at 37^°^C for 2 hours. The products of C3b cleavage were separated by SDS-PAGE under reducing conditions. Proteins were transferred to membranes, immunoblotted with anti-C3 antibody, and visualized by chemiluminescence.

### Biacore analysis

Surface Plasmon Resonance (SPR)-based Biacore binding experiments were performed at 25^o^C on a Biacore 3000 (GE Healthcare). Plasma-purified VWF, recombinant VWF-A1, VWF-A2 or VWF-A3 were immobilized on a CM3 or CM5 sensor chip using amine coupling procedure as recommended by the manufacturer. Briefly, the ligand was diluted in sodium acetate buffer as follows: 40 nM of VWF in 100 mM acetate (pH 5.5), 200 nM of VWF-A1 in 10 mM sodium acetate (pH 5.5), 200 nM of VWF-A2 in 100 mM acetate (pH 5.5), and 200 nM of VWF-A3 in 10 mM acetate (pH 5.5). Following activation of the sensor surface, the protein solution was injected (5 µL/min) and coupled to the surface. The immobilization procedure resulted in a surface density of 2100 RU for VWF, 1500 RU for VWF-A1, 1000 RU for VWF-A2, and 800 RU for VWF-A3. A reference surface with no protein coupled was used as a control. In some experiments, an ovalbumin-coated surface (500 RU) was used as a negative control. HEPES-buffered saline (HBS: 10 mM HEPES, pH 7.3, 150 mM NaCl) was used as running buffer. Plasma purified fH, recombinant full-length, or truncated fH was injected over surface-immobilized ligands, in the presence or absence of different divalent cations. After completion of association and dissociation studies, bound proteins were removed by two-minute injection of HBS containing 3 mM EDTA to regenerate the ligand surface. Baseline-corrected SPR response curves (with buffer blank runs further subtracted) were used for data analysis. Responses at equilibrium of the SPR curves were fitted to a one-site binding (hyperbola) isotherm (GraphPad Prism 4) to obtain equilibrium dissociation constant (*K*_D_). Non-equilibrium data were globally fitted to pre-defined two-state model using BIAevaluation software (Version 4.1). A control experiment was also performed to detect a possible conformational change (linked reaction) during binding of fH to its ligands. In this experiment, 0.5 µM of fH in binding buffer (HBS containing 50 µM of ZnCl_2_) was injected to the ligand surface and allowed to contact for a short (1 min) or a long period of time (4 min). The association rate constant (*k*_a1_) and dissociation rate constant (*k*_d1_) for the binding step, as well as forward rate constant (*k*_a2_) and backward rate constant (*k*_d2_) for the conformational change step were derived from global fitting of the SPR response curve. The apparent dissociation constant (*K*_D_^app^) was calculated according to the formula *K*_D_ = 1/((*k*_a1_/*k*_d1_) * (1+*k*_a2_/*k*_d2_)).

### ELISA assay

Plasma-purified factor H (30 nM) was coated on a 96-well ELISA plate (50 µl each well) at 4^o^C overnight. The coated well was blocked with 1% bovine serum albumin (BSA) for 1 hour at room temperature and washed with PBST. Recombinant VWF-A1 (200 nM), VWF-A2 (200 nM), VWF-A3 (200 nM), PBS alone, or VWF (5 µg/ml) was incubated on immobilized fH (50 µl each well) for 2 hours at room temperature. In some experiments, ristocetin (0.5 mg/ml) was included in the incubation mixture. Following three washes with PBS-T, the fH-bound His-tagged VWF A1, VWF A2, or VWF A3 was detected using a primary anti-His (Penta-His, Qiagen) and secondary HRP-conjugated antibodies in a microplate reader (BioTek) at 450 nm. The fH-bound VWF was detected using a primary anti-VWF and secondary HRP-conjugated antibodies.

### Depleting serum of Factor H or C3

Two hours after preincubation with 50 µl of protein A/G beads, 250 µl of normal pooled serum was centrifuged at 10,000 × *g* for 10 min. The collected serum supernatant was incubated with either 50 µl of polyclonal anti-fH or anti-C3 antibody (complement Technology Inc.) and protein A/G beads for 2 hours at room temperature, and centrifuged to obtain supernatant. C3-depleted serum sample was used as a control for fH-depleted serum in our studies, because fH depletion results in activation of the alternative complement system in few minutes and in a rapid decline in C3 concentration in serum.

### Cleavage of FRETS-VWF73 by ADAMTS-13 in the presence of fH

ADAMTS-13 activity was determined by measuring the rate of cleavage of a fluorogenic substrate that contains 73 amino acids of the A2 domain of VWF (FRETS-VWF73) (Gen-Probe) according to the manufacturer’s protocol. The cleavage reaction in 50 µl of substrate dilution buffer together with 50 µl of test samples (10 nM of ADAMTS-13; 0, 0.4, 0.8, 1.6 µM of fH) was induced by incubation for 30 minutes at room temperature and recorded in a fluorescence reader (BioTek) at excitation and emission wavelengths of 340 nm and 450 nm, respectively. In some of the samples, 100 nM of polyclonal anti-fH antibody was added. Fluorescence measured at time zero (beginning of incubation) was subtracted from values measured after 30 minutes of incubation. ADAMTS-13 activity was determined using a calibration curve based on calibrator standards (Gen-Probe). In another group of experiments, 5 µl of fH- or C3-depleted serum was used to cleave FRETS-VWF73.

For kinetic studies, different concentrations of FRETS-VWF73 (0.5, 1, 2, and 4 µM) were incubated with fixed concentrations of recombinant ADAMTS-13 (10 nM) and fH (0.66 µM) for 30 min at 37^o^C. The V_max__._ was determined by standard calibrator with 100% substrate cleavage activity provided in Gen-Probe kit, and this was used for determining the relative rate of substrate cleavage activity in each sample. The collected data were fitted into a Michaelis-Menten equation, and V_max__._, K*m*, and k_cat__._ were determined by Lineweaver-Burk plot.

### Cleavage of recombinant VWF-A2 by ADAMTS-13 in the presence of fH

We measured the cleavage of recombinant GST-VWF-A2 fusion protein (100 nM) by recombinant ADAMTS-13 (10 nM) in the presence or absence of plasma purified fH (0.6 µM) or recombinant fH (100 nM) in 25 µl of cleavage buffer (100 mM Tris-HCl, pH 8.5, 4 mM CaCl_2,_ 0.02% Brij-35) at 37^o^C. Reactions were quenched by adding reduced sample buffer, and cleaved VWF-A2 was separated by SDS-PAGE on 10% gels and detected by rabbit anti-VWF antibody. The rate of cleavage was quantified by densitometry using NIH ImageJ software (rsb. Info.nih.gov/nih-image). For kinetic studies, we compared the cleavage of VWF-A2 by ADAMTS-13 (10 nM) in the presence or absence of fH (0.6 µM) at different incubation intervals (20 min to 2 hrs) to determine the half-maximal cleavage time.

### HUVEC Culture

The formation of VWF strings anchored to the surface of activated HUVECs was measured under flow conditions as previously described [22]. Briefly, endothelial cells were obtained from human umbilical veins (HUVEC) under a protocol approved by the Institutional Review Board of the Baylor College of Medicine. The umbilical cords were washed with phosphate buffer (140 mM NaCl, 0.4 mM KCl, 1.3 mM NaH_2_PO_4_, 1.0 mM Na_2_HPO_4_, 0.2% glucose, pH 7.4), and then infused with a collagenase solution (0.02%, Invitrogen Life Technologies, Carlsbad, CA). After 30 min incubation at room temperature, the cords were rinsed with 100 ml of the phosphate buffer. Elutes from collagenase-treated umbilical cords were centrifuged at 250 × *g* for 10 min. The cell pellets were resuspended in Medium 199 (Invitrogen Life Technologies) containing 20% heat-inactivated fetal calf serum and 0.2 mM of L-glutamine, and plated on a culture dish coated with 1% gelatin until confluent.

### Parallel-plate flow chamber

HUVECs growing in a 35-mm cell culture dish were stimulated with 25 µM histamine (Sigma-Aldrich, St. Louis, MO) for 10 min at room temperature and then assembled to form the bottom of the parallel-plate flow chamber (Glycotech, Rockville, MD). The assembled chamber was mounted onto an inverted-stage microscope (Nikon, Eclipse TE300, Garden City, NY) and connected to a syringe pump to draw the Tyrode’s buffer containing washed platelets through the chamber at 2.5 dyn/cm^2^ shear stress. The chamber system was kept at 37^o^C with a thermostatic air bath during the experiments. Acquired images were analyzed offline using MetaMorph software (Universal Images, West Chester, PA).

### Cleavage of ultra-large (UL) VWF under flow conditions by ADAMTS-13 in the presence of fH (string Assay)

We measured the cleavage of ULVWF-platelet strings formed on endothelial cells in a parallel platelet chamber as described above. Briefly, HUVECs were stimulated with 25 µM histamine (Sigma Aldrich, St. Louis, MO) for 3 min and then perfused with washed platelets in the presence of recombinant ADAMTS-13 (10 nM, control), ADAMTS-13 with fH (1.3 µM), or ADAMTS-13, factor H, and anti-fH antibody (100 nM) through the parallel-plate flow chamber. The number of platelet-decorated ULVWF strings (20 continuous review fields at 200 x) was counted at 10 S and 2 min time points after perfusion began. ADAMTS-13 activity was defined as the percent reduction of ULVWF strings compared to negative controls of buffer perfusion alone [22].

### Statistics

Data are presented as mean ± SD. Comparisons were made using Student’s *t* test and ANOVA; *p* < 0.05 was considered to be statistically significant.

## Results

### Factor H binds to VWF

**Figure 1:**
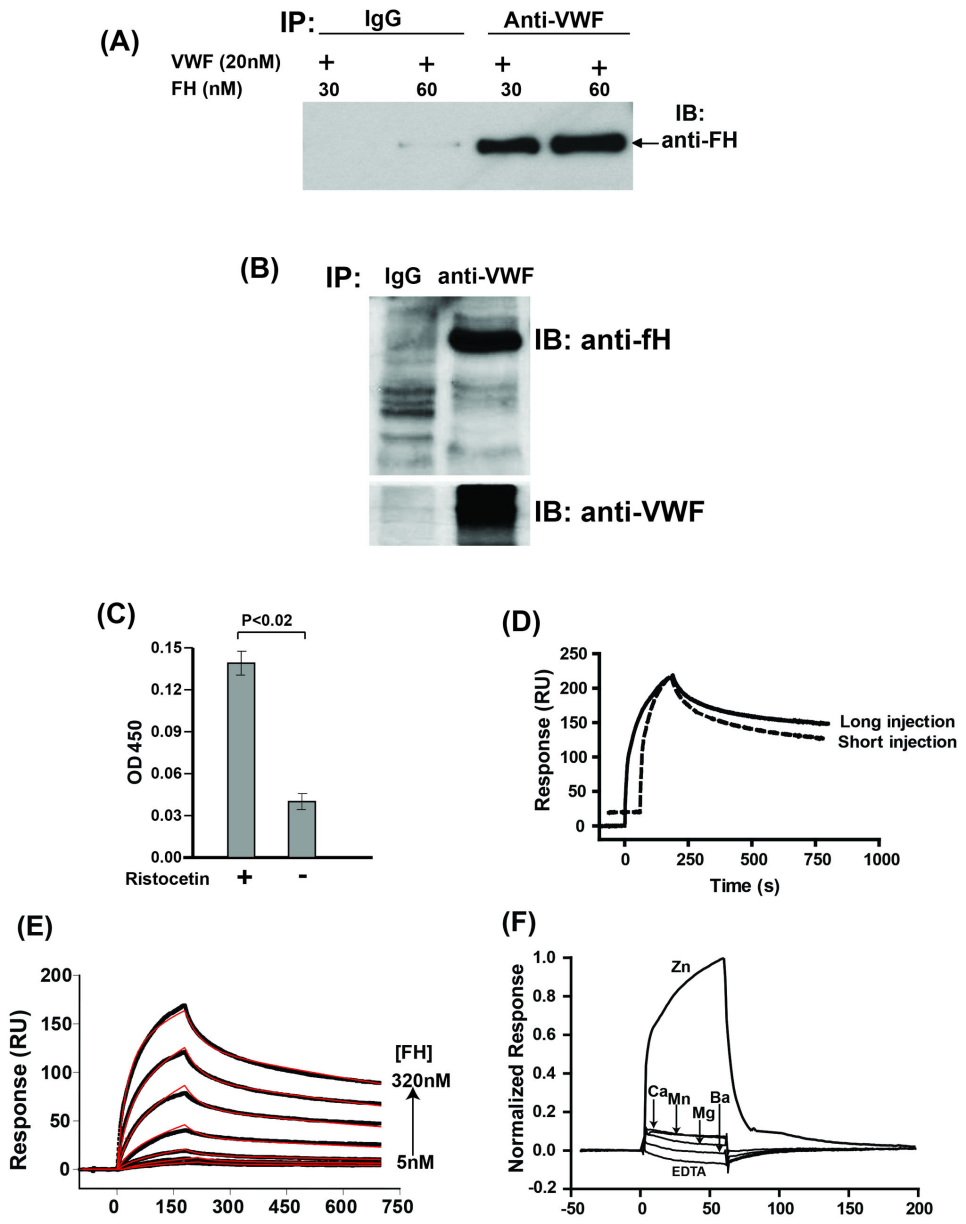
Factor H binds to VWF. (**A**) Purified VWF (20 nM) was incubated with different concentrations of fH (30, 60 nM) and immunoprecipitated (IP) using anti-VWF antibody or control IgG. VWF co-precipitated proteins were immunoblotted using anti-fH antibody. (**B**) Proteins immunoprecipitated from normal human serum using anti-VWF antibody or control antibody was immunoblotted (IB) using anti-fH (upper panel) or anti-VWF antibody (lower panel). A representative blot is shown (n=4). (**C**) Binding of plasma-purified VWF (5 µg/ml) to immobilized fH (30 nM) was studied using ELISA. The results are shown as mean with standard deviation (n=4, p<0.02). (**D**) Dissociation profiles for the fH/VWF interaction using long (solid line) or short (dashed line) injection time of fH (1 µM). Slower dissociation of fH from VWF with a long injection indicated that a conformational change could be involved during binding. (**E**) In a Biacore assay, two-fold increasing concentration (5 to 320 nM) of fH in HBS containing 50 µM ZnCl_2_ were injected over immobilized VWF (40 nM) at a flow rate of 30 µL/min. Baseline corrected SPR response curves (shown in black, with lower curve corresponding to lower concentration of fH injected) were globally fitted to the predefined two-state model. The fitted curves are shown in red and the dissociation constant *K*_D_^app^ of 33 nM was obtained from the fit. (**F**) To study the effect of different cations on binding of fH to VWF, 0.5 µM fH was injected onto the VWF in the presence of HBS running buffer containing ZnCl_2_ (0.1 mM), CaCl_2_ (2 mM), BaCl_2_ (1 mM), MnCl_2_ (1 mM), MgCl_2_ (1 mM), or EDTA (3 mM).

To detect binding of fH to VWF, we coincubated purified VWF and fH in PBS buffer and immunoprecipitated VWF using resin beads coated with anti-VWF antibody. Factor H was detected to coprecipitate with VWF (Figure 1A). We also investigated the association between fH and VWF in serum by studying the proteins co-immunoprecipitated with VWF, and found that fH coprecipitated with VWF in normal serum (Figure 1B); however, we could not determine the extent of this association in serum and determine the fraction of VWF binds to fH *in vivo*. Binding of fH to VWF was enhanced in the presence of ristocetin (Figure 1C). To study the kinetics of the interaction between fH and VWF, we used SPR-based Biacore assay. Binding of full-length fH to immobilized VWF and subsequent dissociation of bound fH were detected as SPR response curve in real time under flow conditions. To identify the correct model for analysis of the binding data, we compared the dissociation phase of the long injection (3 or 4 min) of fH over its ligands with that of the short injection (1 or 2 min) (Figure 1C). The long injection had a slower dissociation than the short injection, indicating a linked reaction during fH interaction with VWF involving conformational changes. Based on these observations, the two state conformational change model was used to fit the sensorgrams and to obtain binding kinetics (Figure 1D). This model describes a 1:1 binding of analyte (fH) to immobilized ligand (VWF or its fragments) followed by a conformational change in the complex. The result showed that a conformational change was involved during the interaction. The association rate constant [*k*_a1_ = 6.0 ± 0.10 (x10^4^ 1/Ms)], dissociation rate constant [*k*_d1_ = 1.58 ± 0.06 (x 10^-2^ 1/s)], forward rate constant [*k*_a2_ = 7.0 ± 0.14 (x 10^-3^ 1/s)], and backward rate constant [*k*_d2_ = 9.0 ± 0.17 (x 10^-4^ 1/s)] were derived from global fitting of SPR response curves (Figure 1D) and the apparent dissociation constant *K*_D_^app^ of 32.9 ± 8.0 nM was determined. This binding affinity was based on the interaction between fH and the surface immobilized VWF, but we could not determine the affinity of this interaction in serum. The effect of different divalent cations (Zn, Ca, Mg, Mn, and Ba) on the interaction between fH and VWF was studied by conducting SPR studies using binding buffer containing these cations. Inclusion of ZnCl_2_ in the binding buffer increased the binding of fH to VWF (Figure 1E). Even though there was a detectable level of fH bound to VWF in the presence of other divalent cations (MnCl_2_, CaCl_2_, BaCl_2_, and MgCl_2_), this amount was only 10% of that in the presence of zinc.

### Binding site of fH on VWF

**Figure 2:**
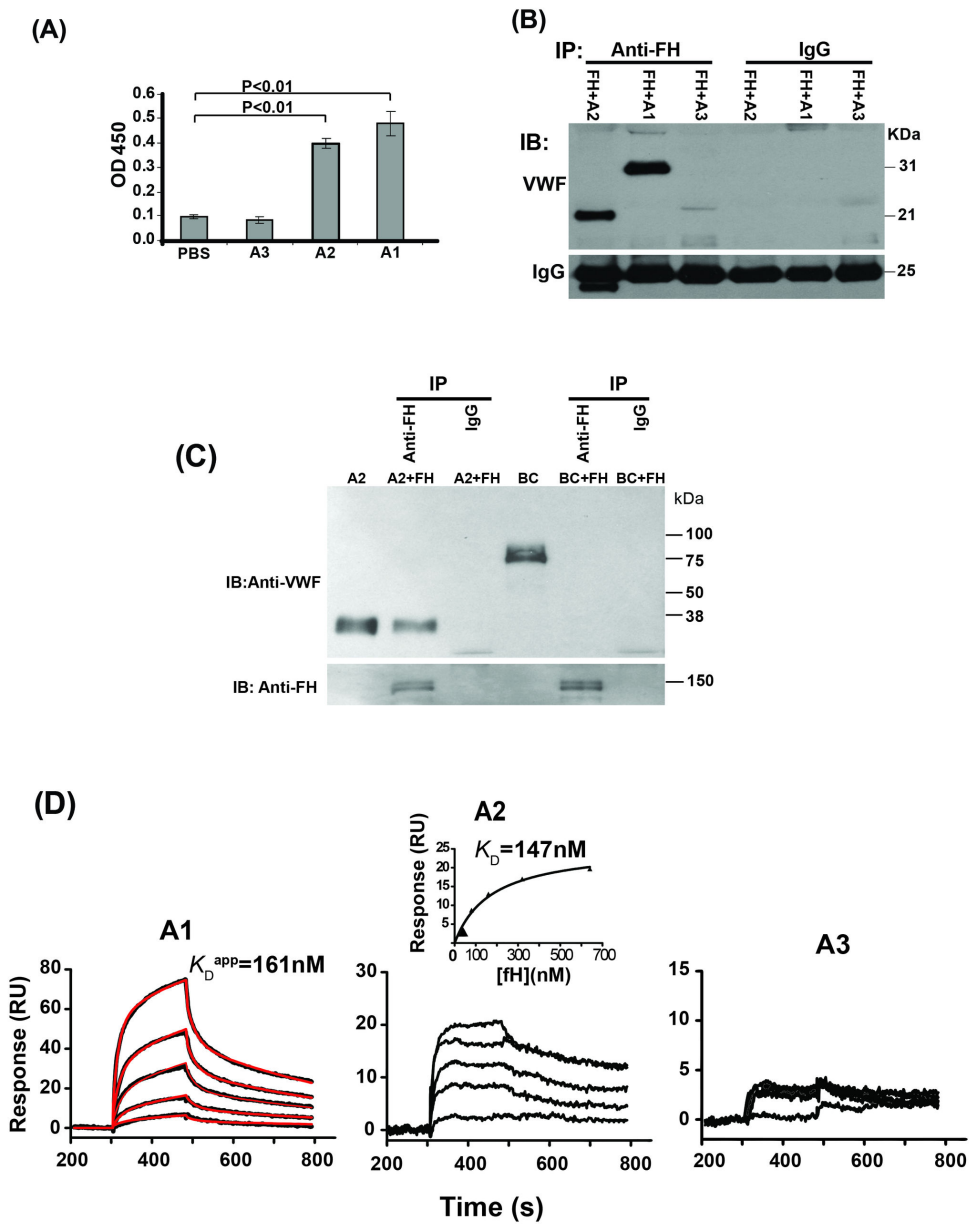
Factor H binds to VWF-A1 and –A2 domains. (**A**) Binding of recombinant fragments of VWF (A1, A2, A3, 200 nM each) to immobilized fH (30 nM) was studied using ELISA. The results are shown as mean with standard deviation (n=3, t-test). (**B**) Recombinant His-tagged VWF-A1, –A2, or –A3 domain (100 nM) was incubated with purified fH (30 nM) and immunoprecipitated (IP) by anti-fH antibody or IgG. Co-precipitated proteins were immunoblotted (IB) with anti-VWF antibody. A representative blot is shown (n=3). (**C**) Recombinant His-tagged VWF-A2 (100 nM) and Myc-tagged VWF-BC domains (100 nM), both purified from transfected HEK 293 cells, were incubated with purified fH (30 nM), precipitated with anti-fH antibody or IgG, separated by SDS- PAGE, and transferred to immobilon-P membranes. The same membrane was blotted with anti-VWF (upper panel) or anti-fH antibody (lower panel). (**D**) In a Biacore assay, two-fold increasing concentration (40 to 640 nM) of fH were injected over immobilized VWF-A1, VWF-A2, or VWF-A3 (200 nM each) at a flow rate of 20 µL/min. Baseline corrected SPR response curves are shown in black, with lower curve corresponding to lower concentration of fH injected. The sensorgrams for fH binding to VWF-A1 were fitted to the predefined two-state model (fitted curves are shown in red). For fH to VWF-A2 binding interaction, the responses at equilibrium of the SPR curves were fitted to a one-site binding isotherm (inset) to obtain the equilibrium dissociation constant (*K*_D_). Binding of fH to VWF-A3 was negligible.

We used ELISA to detect the binding of recombinant VWF-A domains to immobilized fH, and identified binding of VWF-A1 and -A2 domains, but not the VWF-A3 domain, to fH (Figure 2A). To confirm these results, we examined coimmunoprecipitation of fH with recombinant His-tagged VWF-A domains. Factor H coprecipitated with VWF-A1 and VWF-A2 (produced by *E. coli* or mammalian cells) domains but not with the VWF-A3 domain or VWF-BC domains (Figure 2B and 2C). In addition to ELISA and immunoprecipitation, we used SPR to study the binding of fH to VWF-A domains. Factor H was found to bind to immobilized VWF-A1 and -A2 domains; however, binding responses of fH to VWF-A3 were negligible (Figure 2D). Similar to VWF, VWF-A1 domain could also trigger a conformational change in the complex, with similar rate constants (*k*_a1_ = 5.36 x10^4^ 1/Ms, *k*_d1_ = 3.58 x 10^-2^ 1/s, *k*_a2_ = 7.97 x 10^-3^ 1/s, except a faster *k*_d2_ (2.54 x 10^-3^ 1/s) which contributed to a weaker affinity (*K*_D_^app^ = 161 nM). The SPR profiles for fH binding to VWF-A2 were different compared to VWF or VWF-A1; the binding reached equilibrium and could be fitted to a one-site binding isotherm (Figure 2D inset) to obtain equilibrium dissociation constant (*K*_D_ =147 nM).

### Generating recombinant full-length and truncated fH molecules

**Figure 3:**
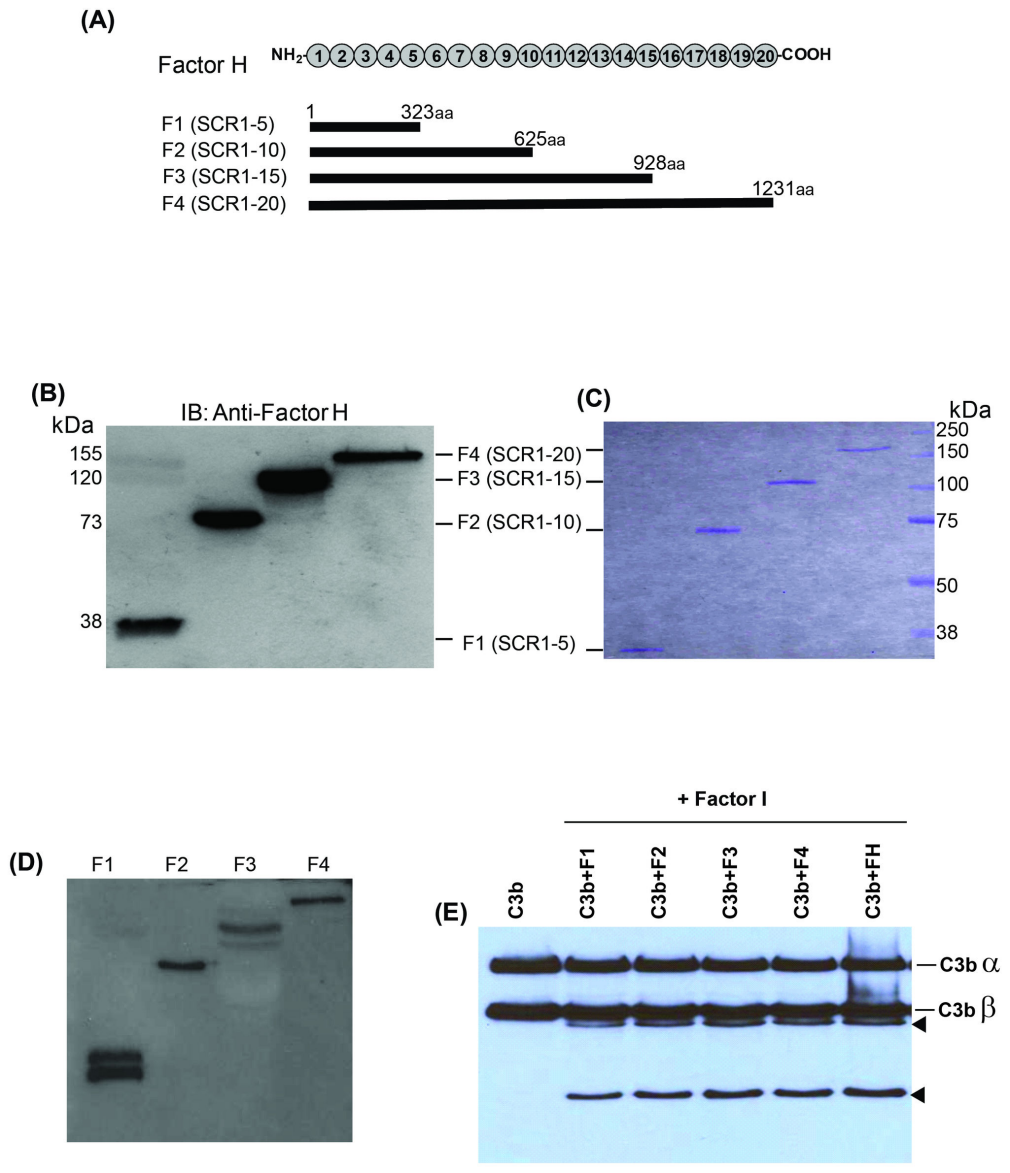
Recombinant full-length and truncated Factor H. (**A**) Schematic representation of fH structure that includes 20 SCR domains and design of recombinant fH molecules used in our experiments. Amino acid lengths and SCR domains of each recombinant fragment are shown. (**B**) Immunoblotting (IB) of purified recombinant full-length and truncated fH molecules using polyclonal anti-fH antibody. (**C**) Purified recombinant full-length and truncated fH molecules were detected after PAGE (10%) and Coomassie blue staining. (**D**) C3b was incubated with plasma purified and recombinant fH molecules and immunoprecipitated using polyclonal anti-C3 antibody. Precipitated proteins were immunoblotted using anti-fH antibody. (**E**) Cofactor activity of plasma purified and recombinant fH molecules was investigated by incubating them with purified C3b and factor I. Factor I-mediated cleavage of C3b generated two cleavage products (arrowheads) originated form C3b α chain.

To identify the region in fH involved in its binding to VWF, we generated recombinant full-length and truncated fH molecules. F4 (includes all 20 SCRs), F3 (SCR1-15), F2 (SCR1-10), and F1 (SCR1-5) are depicted in Figure 3A. The wild type and mutant cDNAs encoding full-length and truncated fH were subcloned into an eukaryotic expression vector and transfected in HEK 293 cell line. The purified expressed proteins are shown after Western-blotting with anti-fH antibody (Figure 3B) and after Coomasie blue staining of the SDS-polyacrylamide gel (Figure 3C). We investigated the structural and functional integrity of the recombinant fH fragments (F1, F2, F3, and F4) by studying their binding to C3b and their Factor I-cofactor activity. All fH recombinant fragments (F1, F2, F3, and F4) contained SCR1-4 and were able to bind to C3b (Figure 3D) and enhanced cleavage of C3b by factor I (Figure 3E).

### Binding site of VWF on fH

**Figure 4:**
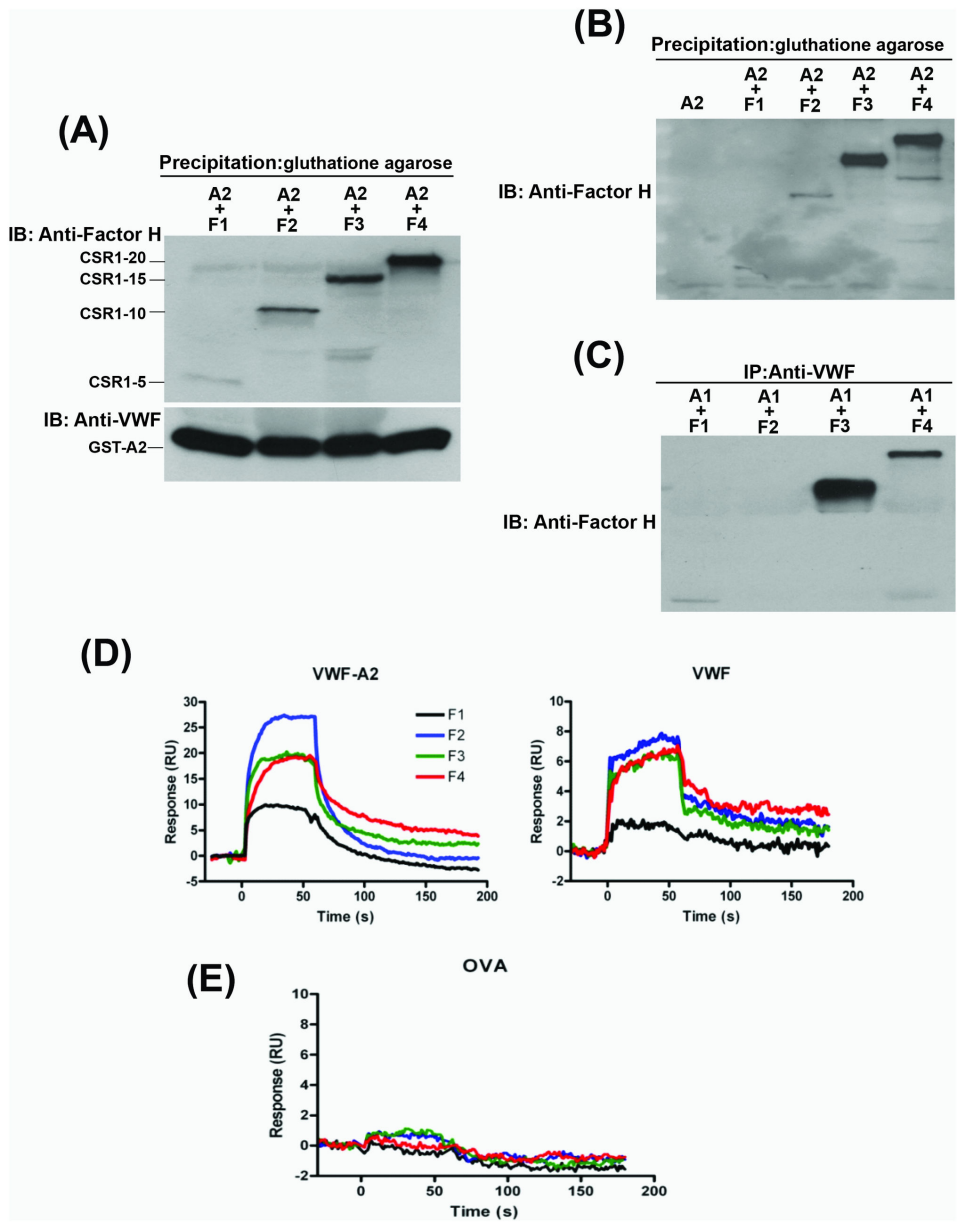
Binding of recombinant full-length and truncated fH molecules to VWF and VWF-A2. (**A**) Recombinant GST VWF-A2 protein (100 nM) was mixed with recombinant full-length or truncated fH (30 nM) and precipitated using glutathione agarose beads. The precipitated proteins were immunoblotted (IB) with anti-fH antibody for examining the association between VWF-A2 and fH molecules. Immunoblotting with anti-VWF antibody served as loading control. A representative blot is shown (n=3). (**B**) We repeated the above experiment but used a monoclonal anti-fH antibody (clone 90X) recognizing SCR1 to immunoblot fH fragments precipitated with GST-VWF-A2. (**C**) Recombinant VWF-A1 protein (100 nM) was mixed with recombinant full-length or truncated fH (30 nM) and precipitated using polyclonal anti-VWF antibody. The precipitated proteins were immunoblotted with monoclonal anti-fH antibody (clone 90X) for examining the association between VWF-A1 and fH molecules. (**D**) SPR sensograms were generated by injecting recombinant fH proteins (F1, F2, F3, or F4) at a concentration of 10 µg/ml over immobilized VWF-A2 or VWF for 60s followed by dissociation for 2 min. (**E**) SPR sensograms generated by injecting fH proteins (F1, F2, F3, and F4) over immobilized ovalbumin (OVA), served as a negative control.

We compared the binding of recombinant VWF-A2 and VWF-A1 to full-length and truncated fH molecules, using a co-immunoprecipitation assay. After incubation with fH molecules, the recombinant VWF-A2 domain (GST-A2 fusion protein) was precipitated using glutathione-conjugated agarose beads. Western blotting with polyclonal anti-fH antibody (Figure 4A) and monoclonal anti-fH antibody recognizing SR1 (Figure 4B) revealed full-length fH (F4) coprecipitating with VWF-A2. Subsequent removal of C-terminal SCRs, F3 (SCR1-15) and F2 (SCR1-10) resulted in a decrease in binding to VWF-A2 compared to full-length fH (F4 with SCR1-20). F1 (SCR1-5) did not bind to VWF-A2 and was not detected among coprecipitated proteins (Figure 4A-B). Immunoprecipitation studies using VWF-A1 and recombinant fH fragments showed that shorter fH fragments (F1 and F2) bound less than longer fragments (F3 and F4) to VWF-A1. These results were consistent with binding of VWF-A1 to SCR10-20 on fH (Figure 4C). To further investigate the binding site of VWF on fH, we used SPR to compare binding of the full-length recombinant fH (F4) to VWF-A2 and VWF with those of truncated fH molecules (F1, F2, and F3). Full-length factor H bound to VWF and VWF-A2 more than truncated fH molecules (Figure 4D). Progressive truncation of fH from the C-terminus resulted in a decrease in binding to VWF-A2 and VWF, so that F1 (SCR1-5) did not show binding to VWF or VWF-A2. Furthermore, in comparing the binding profiles, F1 showed the least stable binding to VWF and VWF–A2 domain, indicated by the fast and complete dissociation of the complex; While full-length fH dissociated slower and remained bound on the chip surface. Binding to ovalbumin served as a negative control and was negligible (Figure 4E).

### Factor H enhances cleavage of VWF

**Figure 5:**
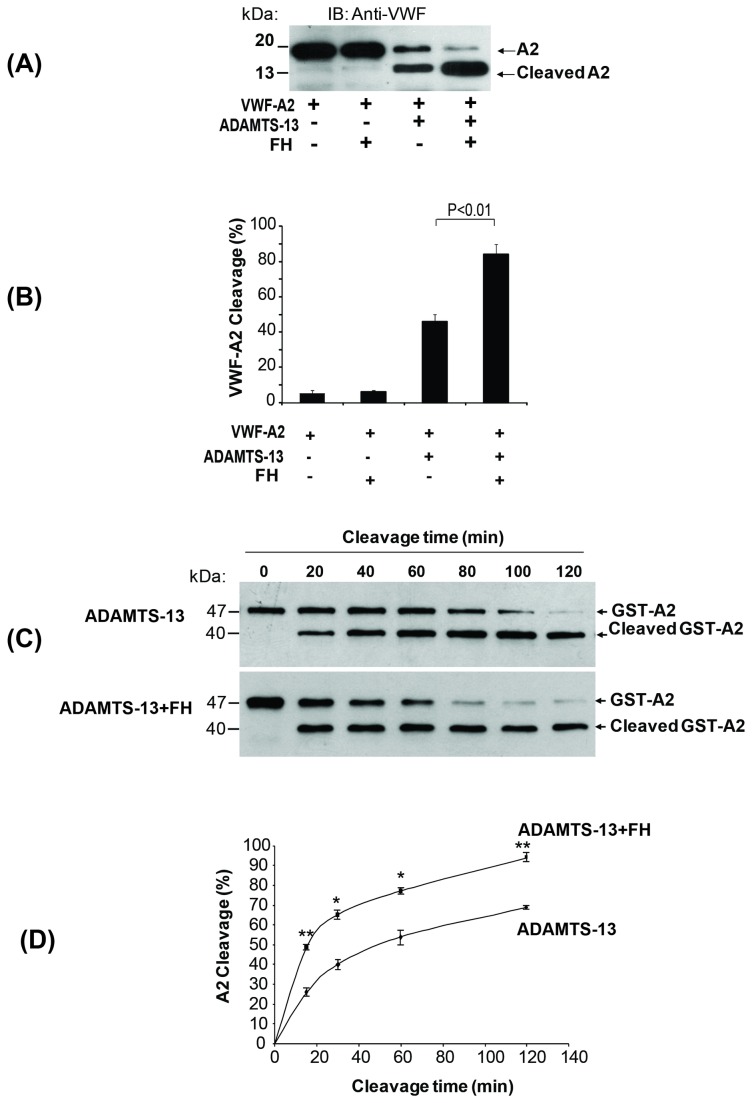
Factor H enhances ADAMTS-13-mediated cleavage of recombinant VWF-A2. (**A**) Cleavage of VWF-A2 (100 nM) by ADAMTS-13 (10 nM) after 1 hr incubation in the presence or absence of fH (0.6 µM) was detected by immunoblotting (IB) with polyclonal anti-VWF antibody. A representative blot is shown (n=5). (**B**) The results of three separate experiments with ADAMTS-13-mediated VWF-A2 cleavage were summarized as bar graphs. The VWF-A2 cleavage (%) was calculated by measuring the ratio of band density of cleaved to uncleaved + cleaved VWF-A2. (**C**) Recombinant GST VWF-A2 (100 nM) was incubated with ADAMTS-13 (10 nM), in the presence or absence of fH (0.6 µM) for different time intervals. The cleavage products were detected by immunoblotting with anti-GST antibody. A representative blot is shown (n=3). (**D**) The half maximal cleavage time of GST VWF-A2 by ADAMTS-13 was calculated using the data obtained from different incubation intervals (20 minutes to 2 hours), in the presence or absence of fH. The results of these experiments were summarized as a linear graph (n=3, t-test, ** p<0.01, * p<0.05).

**Figure 6:**
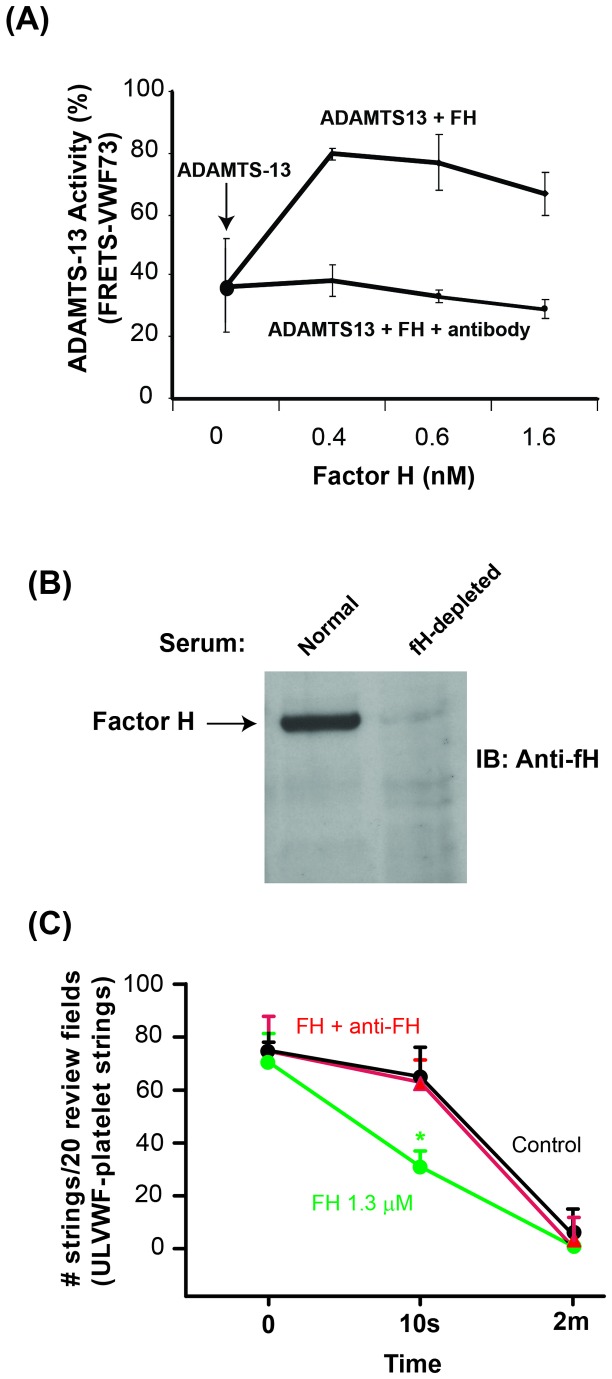
Factor H enhances ADAMTS-13-mediated cleavage of recombinant FRETS-VWF73 and ULVWF. (**A**) Cleavage of ADAMTS-13 substrate (FRETS-VWF73) at different concentrations of fH was quantified using a commercial kit (ATS-13, Gen-Probe). Polyclonal anti-fH antibody (100 nM) was used to block the effect of fH on ADAMTS-13-mediated cleavage of the substrate (n=3). (**B**) Factor H was removed from normal serum by immunoadsorption using sepharose beads coated with polyclonal anti-fH antibody. Immunoblotting of 1 µl of fH-depleted serum with an anti-fH antibody showed the absence of fH band in comparison to normal serum. (**C**) The number of ULVWF strings anchored to the surface of histamine-stimulated-HUVECs (with adherent platelets) in the presence of recombinant ADAMTS-13 (control); ADAMTS-13 and factor H; or ADAMTS-13, factor H and a polyclonal anti-factor H antibody was counted starting at 10 sec or 2 min after perfusion in 20 review fields (×200 magnifications; n=4, *p<0.05).

Cleavage of recombinant VWF-A2 domain after incubation with recombinant ADAMTS-13 was detected by Western-blotting using anti-VWF antibody (Figure 5A). Addition of plasma-purified fH enhanced the cleavage of VWF-A2 by ADAMTS-13 as evident by an increase in the density of cleavage bands. While ADAMTS-13 alone cleaved 46 ± 4% of the VWF-A2 in 60 min; the presence of fH increased the efficiency of cleavage of VWF-A2 to 84 ± 5%, respectively (Figure 5B). For kinetic analyses, we measured the effect of a constant concentration of fH on the cleavage of VWF-A2 at different time points after incubation with ADAMTS-13 (15 min, 30 min, 1 hr, 2 hr, 3 hr, and 4hr), using Western-blotting (Figure 5C). The cumulative results of three separate experiments were summarized in Figure 5D, showing that half-maximal cleavage of VWF-A2 was completed in about 13 min in the presence of fH, compared to about 22 min in the absence of fH. To quantify the effect of fH on ADAMTS-13-mediated VWF cleavage, we measured the activity of recombinant ADAMTS-13 in the presence or absence of plasma-purified fH using FRETS-VWF73 as substrate in a commercial kit (ATS-13, Gen-Probe). Cleavage of the substrate was measured in a fluorometer after 30 min of incubation with recombinant ADAMTS-13. As shown in Figure 6A, plasma-purified fH increased the cleavage of FRETS-VWF73 by recombinant ADAMTS-13 from 36 ± 15% to 79 ± 1% with the effect reaching a plateau at an fH concentration of 0.4µM. Factor H enhancement of ADAMTS-13 activity was completely reversed after the addition of polyclonal anti-fH antibody (Figure 6A). Polyclonal anti-fH antibody alone did not affect ADAMTS-13 activity (data not shown).

We also measured ADAMTS-13 activity in fH-depleted serum. Removal of fH by immunoadsorption (using sepharsoe beads coated with an anti-fH antibody) (Figure 6B) resulted in a 62% reduction in cleavage of FRETS-VWF73 as compared to that in C3-depleted serum (n=3, Data not shown). C3-depleted serum was also prepared by immunoadsorption and served as a control because fH-depletion rapidly results in consumption of serum C3.

We used different concentrations of FRETS-VWF73 substrate to identify the effect of fH on ADAMTS-13 kinetics. We found that the presence of fH enhanced ADAMTS-13-mediated cleavage of FRETS VWF73 by 2.34 folds (K*cat*/K_*m*_ ratio of 164 S^-1^nM^-1^ in the presence of fH and 70.2 S^-1^nM^-1^ in the absence of fH, Table 1).

**Table 1:** Kinetic Cleavage of FRETS-VWF73 by ADAMTS-13 in the presence or absence of fH.

		**Km(μM)**	**k_cat__._ (s^-1^)**	**k_cat_./km (s^-1^nM)**	**Fold change**
ADAMTS-13		5.86 ± 0.94	0.412 ± 0.04	70.2	1.00
ADAMTS-13+ Factor H		12.51 ± 3.78	2.056 ± 0.11	164.4	2.34

The data are expressed as means + SD (n=3).

We then determined whether factor H affected the cleavage of ULVWF strings under flow conditions. Counting the number of strings after 2 min of perfusion showed that the rate of ULVWF cleavage was comparable in the presence or absence of factor H (Figure 6C). However, counting the strings after a shorter perfusion interval (began string counting 10 sec after perfusion started) revealed that factor H significantly accelerated cleavage of ULVWF, an effect that was blocked by preincubation of factor H with a polyclonal Factor H antibody.

### Full-length fH enhances ADAMTS-13 function

**Figure 7:**
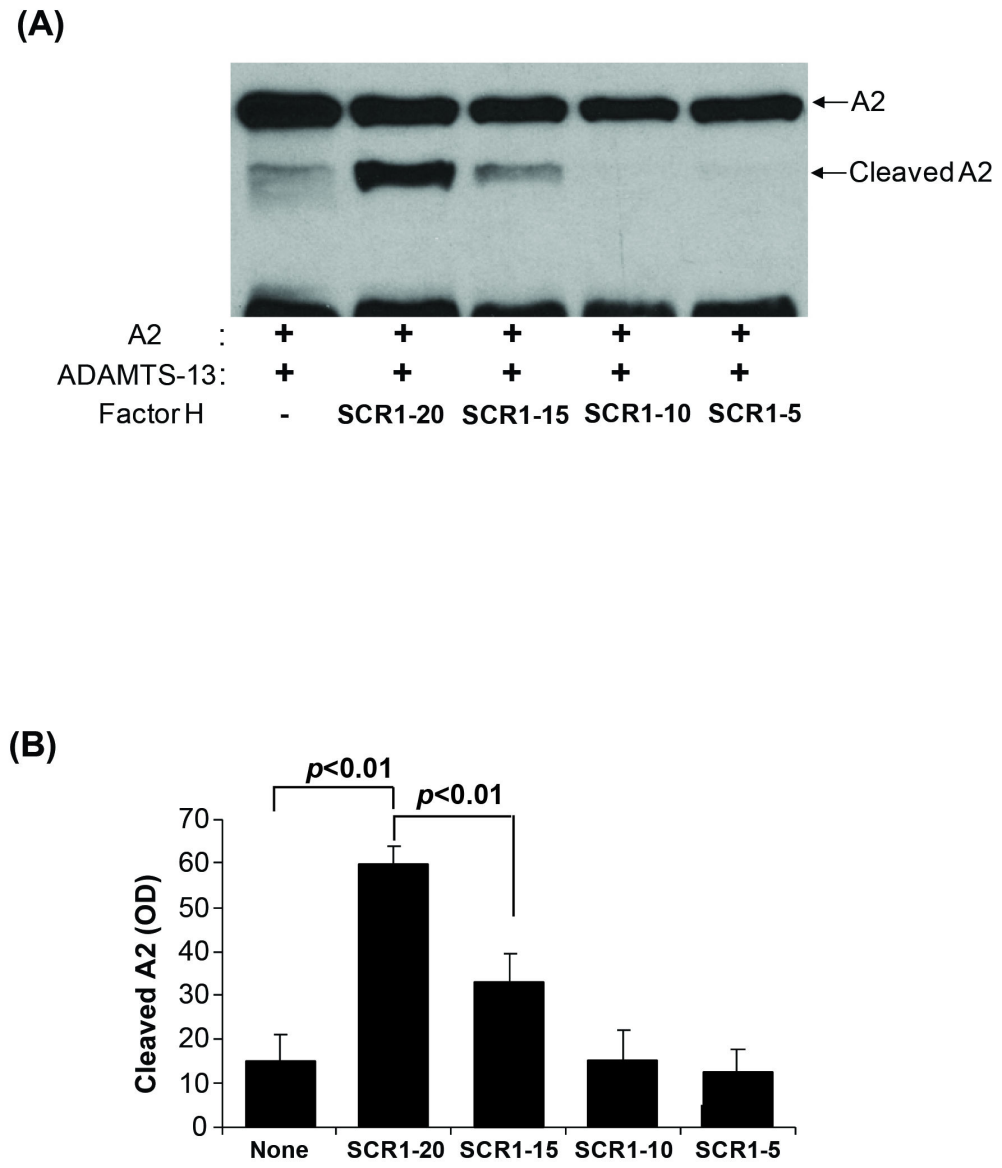
The effect of recombinant full-length and truncated fH on ADAMTS-13-mediated cleavage of VWF-A2. (**A**) Cleavage of recombinant VWF-A2 (100 nM) after 30 minutes of incubation with ADAMTS-13 (10 nM) in the presence or absence of full-length or truncated fH (100 nM) was studied by Western-blotting using anti-GST antibody. A representative blot is shown (n=7). (**B**) The band intensity of the VWF-A2 cleavage products was compared between samples with full-length and truncated fH (n=7, t-test).

We compared the effect of full-length and truncated fH molecules on ADAMTS-13-mediated cleavage of VWF-A2 (Figure 7A). Removal of C-terminal SCRs progressively resulted in a decrease in the fH enhancement of VWF-A2 cleavage. F3 (lacking SCRs 15-20) showed reduced enhancement of cleavage compared to the full-length fH (F4) molecule, while F2 and F1 (missing SCRs 10-20 and 5-20, respectively) showed no enhancement of cleavage (Figure 7B).

## Discussion

Factor H binds to glycosoaminoglycans on the cell membrane and protects cells against complement-induced damage. It binds to several other ligands, including heparin, C3b, integrins α_v_β_3_ and α_IIb_β_3_, and C-reactive protein [23–26], but the functional consequences of these protein–protein interactions are not clear. In this study, we identified VWF as an additional protein partner for fH. We demonstrated a high affinity interaction (*K*_D_^app^ ~ 33 nM) between these two multidomain proteins. This is a high affinity and one would predict that all VWF multimers circulating in the blood are fH-bound. However, this is unlikely to be the case for three reasons. First, binding affinities were calculated from the binding of fH to either immobilized VWF multimers or recombinant A1 and A2 domains *in vitro*. In both experimental conditions, the A1 and A2 domains are exposed and readily available for fH to bind to. Normally, both domains are buried in the tertiary structure of circulation VWF multimers, inaccessible to fH. Therefore, the amount of circulating VWF multimers that is fH-bound is expected to be small in a non-disease state. This notion is supported by the report by Hulstein et al. [27] that a nanobody made against the VWF A1 detected a very small amount of VWF in blood samples from healthy subjects, but a significantly increased amount of VWF in patients with TTP. It is also supported by our finding that the VWF-fH interaction was enhanced in the presence of ristocetin, which is known to induce an active conformation of VWF [21,28]; and is consistent with a recent report by Turner et al., who showed binding of fH to ULVWF strings secreted by, and anchored to, endothelial cells [29]. Second, VWF exists in the blood as multimers of different sizes and a calculated binding affinity for such a heterogeneous ligand may be accurate only for VWF of a particular size. Finally, in our studies binding affinities were measured with purified proteins without likely interferences from other plasma proteins.

We showed that fH binding to VWF increases ADAMTS-13-mediated cleavage of VWF-A2. This might be due to a conformational change in VWF induced by binding of fH to VWF-A2 that makes the cleavage site more accessible to ADAMTS-13. In order to identify the region(s) of fH involved in its binding to VWF, we generated recombinant full-length and truncated fH molecules. Removal of C-terminal SCRs decreased binding of fH to VWF. Progressive truncations of fH C-terminal SCRs progressively diminished its binding to VWF and VWF-A2, until fH truncated of its 15 C-terminal SCRs (SCR 1-5 intact) was finally unable to bind to VWF and VWF-A2. The decrease in binding of truncated fH to VWF was associated with a decrease in its effect on ADAMTS-13-mediated cleavage of VWF-A2. The presence of SCRs 15-20 in fH was important for its cofactor role in VWF cleavage, and fH fragments without this region (F3, F2, and F1) had less effect on ADAMTS-13-mediated VWF-A2 cleavage. In fact, removal of SCRs 10-20 caused a complete elimination of fH’s effect on VWF-A2 cleavage. Together, these results suggest that fH modifies VWF cleavage primarily by binding and by potentially making VWF more accessible to ADAMTS-13.

This functional interaction between fH, VWF and ADAMTS-13 could contribute to the pathogenesis of thrombotic microangiopathy in both TTP and aHUS and provide a mechanistic basis for the frequently observed clinical overlap between TTP and HUS. Mutations in fH are the most common genetic alteration detected in patients with aHUS [9,30] and most of these mutations are located in SCRs 18-20, encompassing the region of fH directing cell surface binding [5–8,10]. The mechanism of thrombotic microangiopathy in aHUS is not known, but is probably related to complement-induced damage resulting from lack of negative regulation of the complement system. Four observations provide evidence in support of this concept. First, mutations in other complement regulatory proteins such as CD46 or factor I cause aHUS [30,31]. Second, aHUS developed in fH deficient mice only after restoration of normal plasma C3 concentrations by the transgenic expression of truncated fH molecule (SCRs 1-15), which is capable of modulating complement activation in plasma but not on cell surfaces [14]. Third, in this transgenic mouse model, aHUS could be reversed when C5 deficiency was induced [32]. Four, inhibition of the complement system using a monoclonal anti-C5 antibody (Eculizumab, Alexion Pharmaceuticals) was successful in treating patients with aHUS [33–35].

Our data introduce the hypothesis that fH may contribute to clinical thrombotic microangiopathies through a complement-independent mechanism. We demonstrated that full-length fH enhances cleavage of VWF-A2 by ADAMTS-13 while fH molecules truncated of regions affected by aHUS-inducing mutations lose this capacity. This hypothesis is consistent with reports that ADAMTS-13 activity was significantly lower in the acute phase of recurrent or familial HUS [15,16].

In TTP, deficiency in ADAMTS-13 function is the main etiologic factor. However, reports of some patients with a clinical diagnosis of TTP without severe ADAMTS-13 deficiency [36], and the presence of a few individuals with severe ADAMTS-13 deficiency who either do not develop TTP or do so only later in life [37,38], suggest that other molecules (as co-factors) may be involved in the pathogenesis of TTP. We speculate that low levels of fH act as a cofactor to ADAMTS-13 deficiency in the pathogenesis of TTP.

In conclusion, we have shown that fH binds to VWF and enhances its cleavage by ADAMTS-13. This interaction could be a component in the pathogenesis of aHUS and it provides a mechanistic link between the pathophysiology and clinical manifestations of thrombotic microangiopathies [39]. Further work to characterize the structural and functional elements directing this interaction should elucidate molecular mechanisms of the thrombotic microangiopathic syndromes and could provide new approaches to the diagnosis and treatment of these dangerous diseases.
